# Subarachnoid haemorrhage with negative initial neurovascular imaging: a systematic review and meta-analysis

**DOI:** 10.1007/s00701-019-04025-w

**Published:** 2019-08-13

**Authors:** Midhun Mohan, Abdurrahman I. Islim, Fahid T. Rasul, Ola Rominiyi, Ruth-Mary deSouza, Michael T. C. Poon, Aimun A. B. Jamjoom, Angelos G. Kolias, Julie Woodfield, Krunal Patel, Aswin Chari, Ramez Kirollos, British Neurosurgical Trainee Research Collaborative

**Affiliations:** 1 Department of Neurosurgery, The Walton Centre NHS Foundation Trust and University of Liverpool, Liverpool, UK; 2 Department of Neurosurgery, Queen’s Hospital, Romford, UK; 3 Department of Neurosurgery, Sheffield Teaching Hospitals NHS Foundation Trust, Sheffield, UK; 4 Institute of Neurology, University College London, London, UK; 5 Usher Institute, University of Edinburgh, Edinburgh, UK; 6 Department of Clinical Neurosciences, Western General Hospital, Edinburgh, UK; 7 Division of Neurosurgery, Department of Clinical Neurosciences, University of Cambridge and Addenbrooke’s Hospital, Cambridge, UK; 8 Division of Neurosurgery, Krembil Research Institute, Toronto Western Hospital, University Health Network and University of Toronto, Toronto, Canada; 9 Institute of Child Health, University College London, London, UK; 10 Department of Neurosurgery, Great Ormond Street Hospital, Great Ormond Street, WC1N 3JH London, UK

**Keywords:** Meta-analysis, Non-aneurysmal, Subarachnoid haemorrhage, Systematic review

## Abstract

**Background:**

In patients with spontaneous subarachnoid haemorrhage (SAH), a vascular cause for the bleed is not always found on initial investigations. This study aimed to systematically evaluate the delayed investigation strategies and clinical outcomes in these cases, often described as “non-aneurysmal” SAH (naSAH).

**Methods:**

A systematic review was performed in concordance with the PRISMA checklist. Pooled proportions of primary outcome measures were estimated using a random-effects model.

**Results:**

Fifty-eight studies were included (4473 patients). The cohort was split into perimesencephalic naSAH (PnaSAH) (49.9%), non-PnaSAH (44.7%) and radiologically negative SAH identified on lumbar puncture (5.4%). The commonest initial vascular imaging modality was digital subtraction angiography. A vascular abnormality was identified during delayed investigation in 3.9% [95% CI 1.9–6.6]. There was no uniform strategy for the timing or modality of delayed investigations. The pooled proportion of a favourable modified Rankin scale outcome (0–2) at 3–6 months following diagnosis was 92.0% [95% CI 86.0–96.5]. Complications included re-bleeding (3.1% [95% CI 1.5–5.2]), hydrocephalus (16.0% [95% CI 11.2–21.4]), vasospasm (9.6% [95% CI 6.5–13.3]) and seizure (3.5% [95% CI 1.7–5.8]). Stratified by bleeding pattern, we demonstrate a higher rate of delayed diagnoses (13.6% [95% CI 7.4–21.3]), lower proportion of favourable functional outcome (87.2% [95% CI 80.1–92.9]) and higher risk of complications for non-PnaSAH patients.

**Conclusion:**

This study highlights the heterogeneity in delayed investigations and outcomes for patients with naSAH, which may be influenced by the initial pattern of bleeding. Further multi-centre prospective studies are required to clarify optimal tailored management strategies for this heterogeneous group of patients.

**Electronic supplementary material:**

The online version of this article (10.1007/s00701-019-04025-w) contains supplementary material, which is available to authorized users.

## Introduction

Spontaneous subarachnoid haemorrhage (SAH) is a life-threatening condition most commonly caused by the rupture of an intracranial aneurysm [18, 49]. Once SAH has been confirmed, dedicated cerebrovascular imaging is performed to establish if there is an underlying vascular abnormality. CT angiography (CTA) is the most common initial cerebrovascular imaging modality used in the UK and Ireland [22], although digital subtraction angiography (DSA) is considered the gold standard [79]. Early detection of vascular abnormalities is important to direct timely treatment [23].

In up to 15% of patients with spontaneous SAH, a structural cause for the bleeding is not identified on initial vascular imaging [23], and these are said to have experienced a non-aneurysmal SAH (naSAH). This group is heterogeneous and can be categorised into three subgroups, based on the distribution of blood on the initial non-contrast CT scan [70]:

- Perimesencephalic (PnaSAH): Defined as “blood present anterior to the midbrain with or without extension to the anterior ambient cistern or basal Sylvian fissure and without complete filling of the interhemispheric fissure or extension to the lateral Sylvian fissure”.
- Non-perimesencephalic (non-PnaSAH): Acute blood is seen on the CT scan that is not confined to the PnaSAH territory.
- Radiologically negative: No acute blood on the CT scan. Diagnosis is made by spectrophotometric detection of a bilirubin peak in cerebrospinal fluid from a lumbar puncture (LP).

Although commonly accepted that patients with naSAH experience a “better” clinical course than aneurysmal SAH patients [23, 76], there is uncertainty regarding the functional outcomes and complications in these patients.

There also appears to be a wide variation in the investigation, follow-up and delayed pick-up rate of vascular abnormalities in naSAH patients [55]. This is particularly important since, although DSA is the gold-standard investigation, it represents an invasive procedure that is not without risk of complications [16]. Furthermore, subjecting a patient to multiple DSAs needs to be weighed carefully against the benefits and probability of identifying a cause for the bleed. Since CTA has become the first-line investigation, the added value of conventional DSA in these patients has also not been firmly established [29].

## Objectives

This systematic review and meta-analysis aimed to summarise the literature on the investigation and management of naSAH by:

i. Synthesising estimates of complications risk and functional outcomes reported to date.
ii. Collating and comparing the various neurovascular imaging strategies and delayed diagnoses rates reported for this cohort of patients.

## Methods

This systematic review was registered with PROSPERO (CRD42016035376) and has been reported in accordance with the PRISMA (Preferred Reporting Items for Systematic Reviews and Meta-Analyses) statement [46].

### Search strategy

Medline, Web of Knowledge, Embase and the Cochrane Database of Systematic Reviews, via the National Institute’s for Health and Care Excellence Health Databases Advanced Search (NICE HDAS) function, were inspected. The search strategy utilised was as follows:

i. (“non aneurysmal” OR “non-aneurysmal” OR “perimesencephalic” OR “angiogram-negative” OR “angiogram negative”).
ii. (“subarachnoid hemorrhage” OR “subarachnoid haemorrhage” OR “SAH”).
iii. i AND ii.

The bibliographies of accepted papers were examined for additional articles not identified in the initial search. The search was last updated in April 2018.

### Study selection

The resulting titles and abstracts were screened independently by two authors (M.M. and O.R.) using the patients, intervention, comparator, outcomes and study design (PICOS) criteria below. All decisions about an article’s inclusion or exclusion were blinded. Where disagreements occurred, a senior author (A.C.) was consulted.

### PICOS criteria

- Patients: Adults (≥ 16 years) with a diagnosis of spontaneous SAH (either by CT brain or LP) and negative initial neurovascular imaging. The term “initial vascular imaging” was defined as one or more dedicated cerebrovascular imaging techniques (CTA, magnetic resonance angiography [MRA] or DSA) performed within 72 h of ictus. If no time-period was specified in the study, but the study stated, “initial imaging”, this was taken as the form of initial imaging strategy used. Traumatic SAH was excluded.
- Interventions/Comparators: Not required.
- Outcomes:

i. Primary: Functional outcomes at 6 months post-ictus.
ii. Secondary: Functional outcomes at discharge and ≥ 1 year post-ictus, delayed diagnoses (including timing and modality of imaging that detected the abnormality), complications (re-bleeding, hydrocephalus, vasospasm, seizure or serum sodium (Na^+^) abnormality [hypo- and hypernatremia]), length of hospital stay, cost and patient perception of information provided by clinical team. Delayed diagnosis was defined as the diagnosis of an aneurysm or any other vascular abnormality that could explain the initial bleed, not identified by the initial vascular imaging strategy but subsequently identified on repeat imaging.

- Study design: Full-text English-language publications of studies with ≥ 10 patients.

### Data extraction

Demographics data, presenting bleeding pattern (PnaSAH, non-PnaSAH, radiologically negative) and initial neurovascular imaging strategies were extracted independently by two authors (M.M. and O.R.) using a standardised proforma, with disagreements resolved via further review and discussion with a senior author (A.C.).

### Data synthesis

#### Presenting clinical-radiological status

The initial clinical grade recorded either using the World Federation of Neurosurgical Societies (WFNS) or Hunt and Hess grading system, and radiological grade via Fisher or the modified Fisher grading system were noted. For pooled analyses, both clinical measures were dichotomised into good (grades 1–3) and poor (grades 4–5) and modified Fisher grades were translated back into the original grading system (Modified Fisher 0 ➔ Fisher 1, 1 ➔ 2, 3 ➔ 3, 2 & 4 ➔ 4).

#### Functional outcomes

These were examined at three time-points—at discharge (or early outcome), 3–6 months and at ≥ 1 year. Outcomes were dichotomised into favourable (modified Rankin scale [mRS] 0–2) and unfavourable (mRS 3–6).

#### Post-naSAH complications

Re-bleeding was split into early (prior to discharge or < 14 days) and late based on the common definitions used by the included studies. Hydrocephalus requiring surgical intervention was categorised into temporary (requiring temporary external ventricular drainage only) and long term (requiring CSF shunting). Vasospasm was sub-divided into radiological and clinical (i.e. delayed ischemic neurological deficit [DIND]).

### Statistical analysis

Study-level data were collated and presented as number (percentage), mean (standard deviation [SD]) or median (interquartile range [IQR]) as appropriate. For each outcome of interest, the primary summary statistic extracted from the included studies was the incidence risk or proportion (number of events/total number of patients). To obtain an overall summary statistic (95% confidence interval [95% CI]), the following steps were followed:

i. The proportions of the included studies were transformed using the Freeman-Tukey double arcsine method [26]. This allowed for normalisation and variance stabilisation of data with a binomial distribution and enabled the use of studies with null event rates as to represent the whole study population.
ii. Transformed proportions were combined using an inverse-variance weighted random-effects meta-analysis model as described by DerSimonian and Laird [19]. For ease of interpretation, the aggregated number of the transformed proportions was converted back to the original scale (inverse-variance). To account for suspected methodological variation and population diversity, a random-effects model, which assumes unequal variance between studies, was used to distribute statistical weighting more conservatively; small studies were not discounted, and larger studies were less likely to dominate the analysis.

Variation across studies, owing to heterogeneity rather than chance, was estimated using the *I*^2^ statistic and categorised into low (≤ 25%), moderate (~ 50%) and high (≥ 75%). SPSS ® v24.0 and StatsDirect ® v3.0 were used for statistical analyses.

### Quality assessment

The NIH Quality Assessment Tool for Observational Cohort and Cross-Sectional Studies was used to assess risk of bias (Fig. e-1). All quality assessments were undertaken in a blinded fashion by two authors (M.M. and A.I.I.). Where discrepancies arose, a third author (A.C.) was consulted.

## Results

**Fig. 1:**
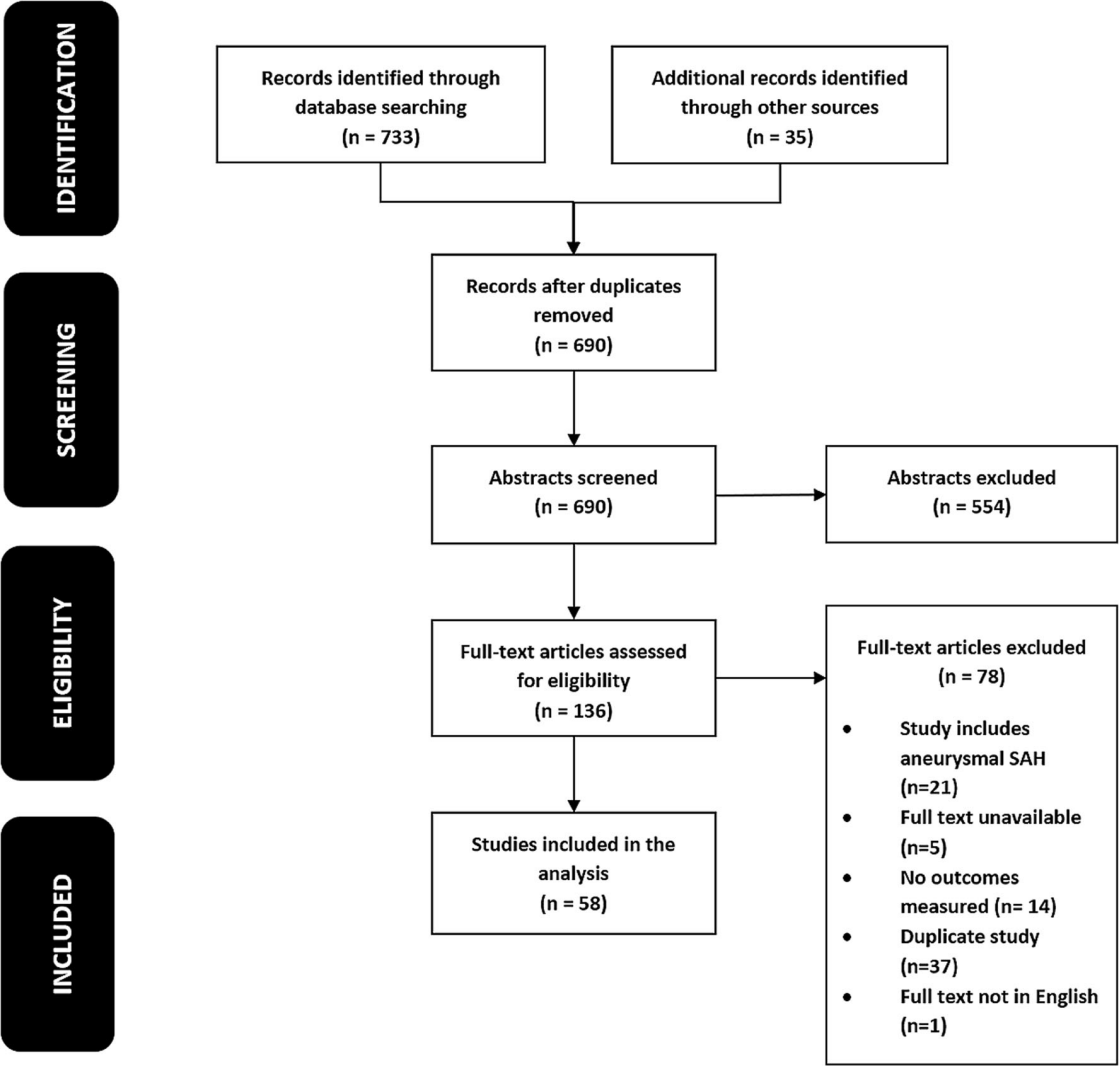
PRISMA flow chart

A total of 58 studies were included, totalling 4473 patients (Fig. 1). Of these, 19 were prospective [1, 2, 9, 10, 20, 21, 32, 38, 39, 41–44, 47, 48, 50, 51, 53, 54], 37 were retrospective [4, 6–8, 12–15, 17, 24, 25, 27, 28, 30, 31, 34–36, 40, 57, 58, 61, 63, 66–69, 71–76, 78, 80–82] and two studies [11, 52] had both prospective and retrospective data (Table e-1). Quality assessment results are summarised in Fig. e-2. Three studies (5.17%) were “poor”, 31 (53.4%) were “fair” and 24 (41.4%) were “good”.

### Demographics and pattern of bleeding

Among the 49 studies (3853 patients) in which demographic data were reported, the weighted mean age of included subjects was 53.8 years. Forty-five studies (3493 patients) reported sex and 54.3% were male (Table e-1). Forty-five studies (3530 patients) sub-classified naSAH by pattern of bleeding; 49.9% (1763/3530) patients had a PnaSAH, 44.7% (1577/3530) patients had a non-PnaSAH and 5.4% (190/3530) had radiologically negative SAH.

**Fig. 2:**
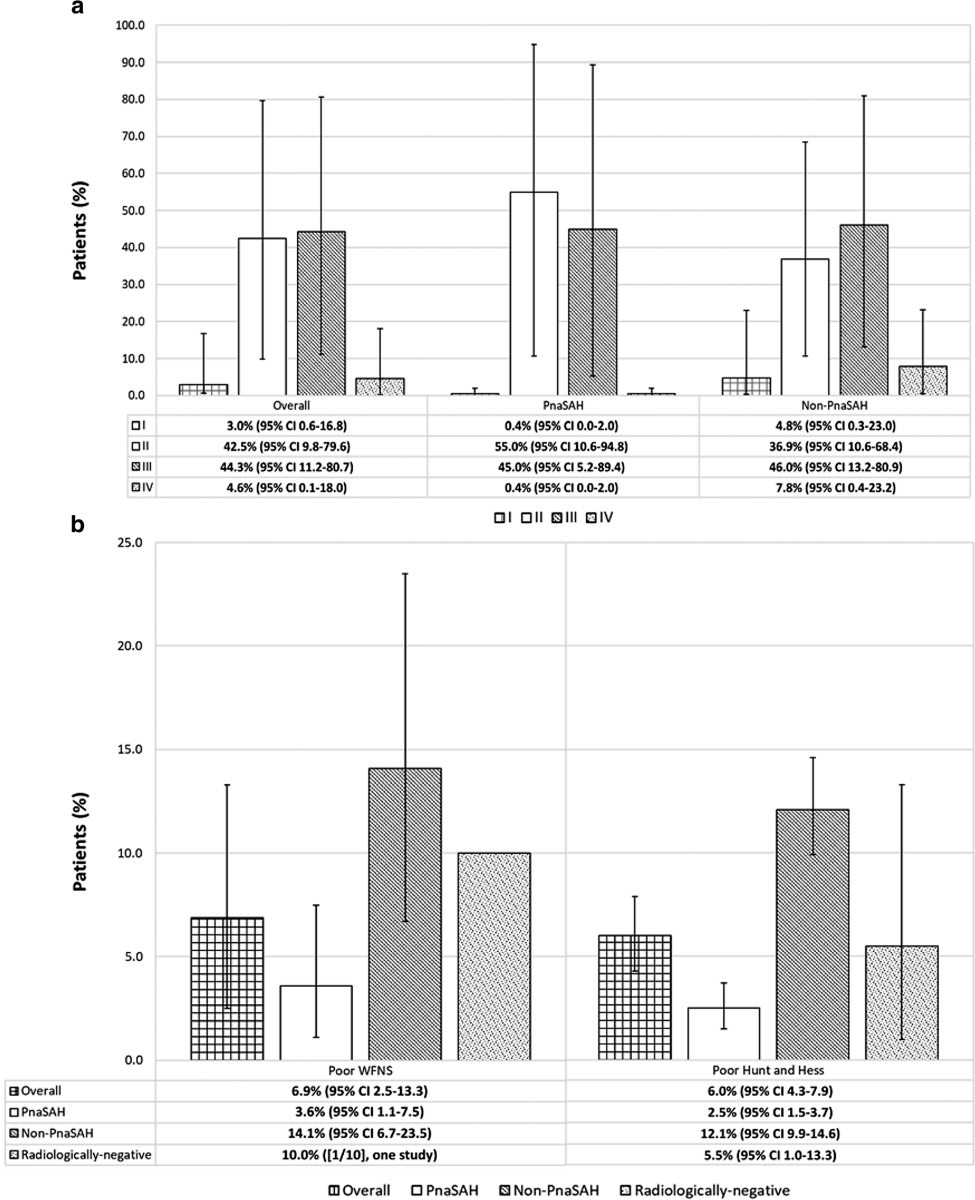
**a** Presenting Fisher grade stratified by bleeding pattern. Pooled percentages were informed by three studies (350 patients). **b** Presenting WFNS and Hunt and Hess grades stratified by bleeding pattern. Pooled WFNS percentages were informed by nine studies (594 patients). Pooled Hunt and Hess percentages were informed by 19 studies (1545 patients)

Pooled proportions of presenting WFNS grade, Hunt and Hess grade and Fisher grade are shown in Fig. 2. A higher proportion of non-PnaSAH patients had poor presenting clinical and radiological grade.

### Initial vascular imaging and delayed diagnoses

The initial vascular imaging strategies, reported in 50 studies, were heterogenous—DSA only (64%), CTA + DSA (16%), DSA + MRA (6%), CTA + DSA + MRA (6%), CTA only (4%) and DSA or CTA (4%).

**Fig. 3:**
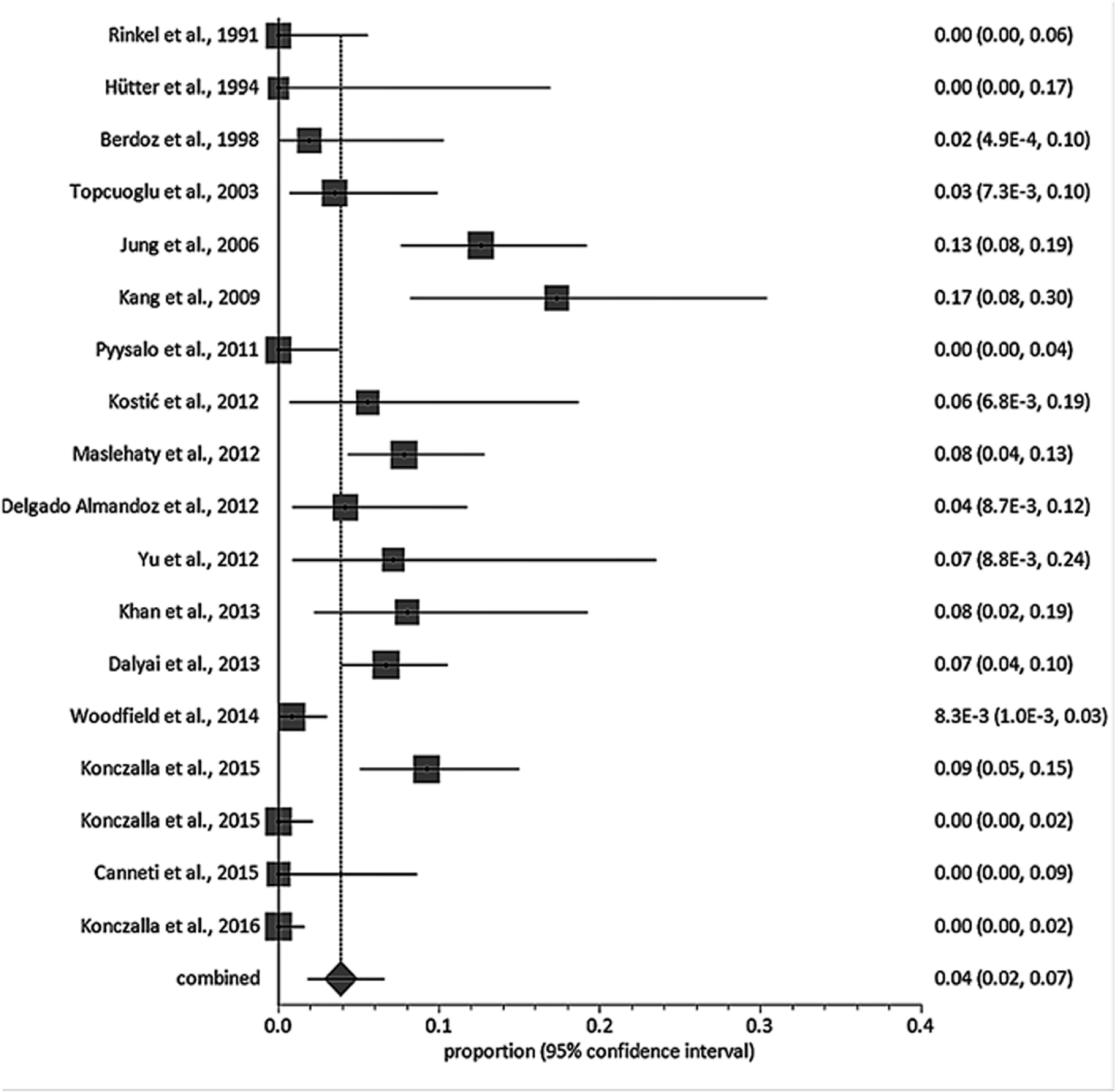
Forest plot showing the risk of delayed diagnosis (95% CI). Horizontal lines denote 95% CIs; solid squares represent the point estimate of each study and the diamond represents the pooled proportion of delayed diagnoses (3.9% [95% CI 1.9–6.6, *I*^2^ = 85.6%]). The size of the solid squares is proportional to the weight of the study

Eighteen studies reported delayed diagnoses data. From a total of 1965 patients, 89 patients were found to have a vascular abnormality at a delayed investigation (range from 0 to 17.3%; Table e-2), yielding a pooled proportion of 3.9% [95% CI 1.9–6.6, *I*^2^ = 85.6%] (Fig. 3). Details regarding the timing of the delayed imaging are available in Table e-2; timing was not reported in 14/18 (77.8%) studies and the earliest and latest investigations in the four other studies were reported at ≤ 72 h and ≥ 1 year from ictus. Anterior circulation aneurysm (60.6%) was the most commonly reported diagnosis (Table 1).

**Table 1:** Delayed diagnoses details available for 66 patients

Diagnosis	*N* (%)
Anterior circulation aneurysm	40 (60.6)
Posterior circulation aneurysm	15 (22.7)
Aneurysm^a^	2 (3.0)
Posterior circulation pseudoaneurysm	2 (3.0)
Infundibulum^a^	2 (3.0)
Spinal AVM^a^	1 (1.5)
Brain AVM^a^	1 (1.5)
Brain cavernoma^a^	1 (1.5)
Vertebral artery dissection	1 (1.5)
AV fistula^a^	1 (1.5)

*AV* arteriovenous, *AVM* arteriovenous malformation

^a^Location unspecified

**Fig. 4:**
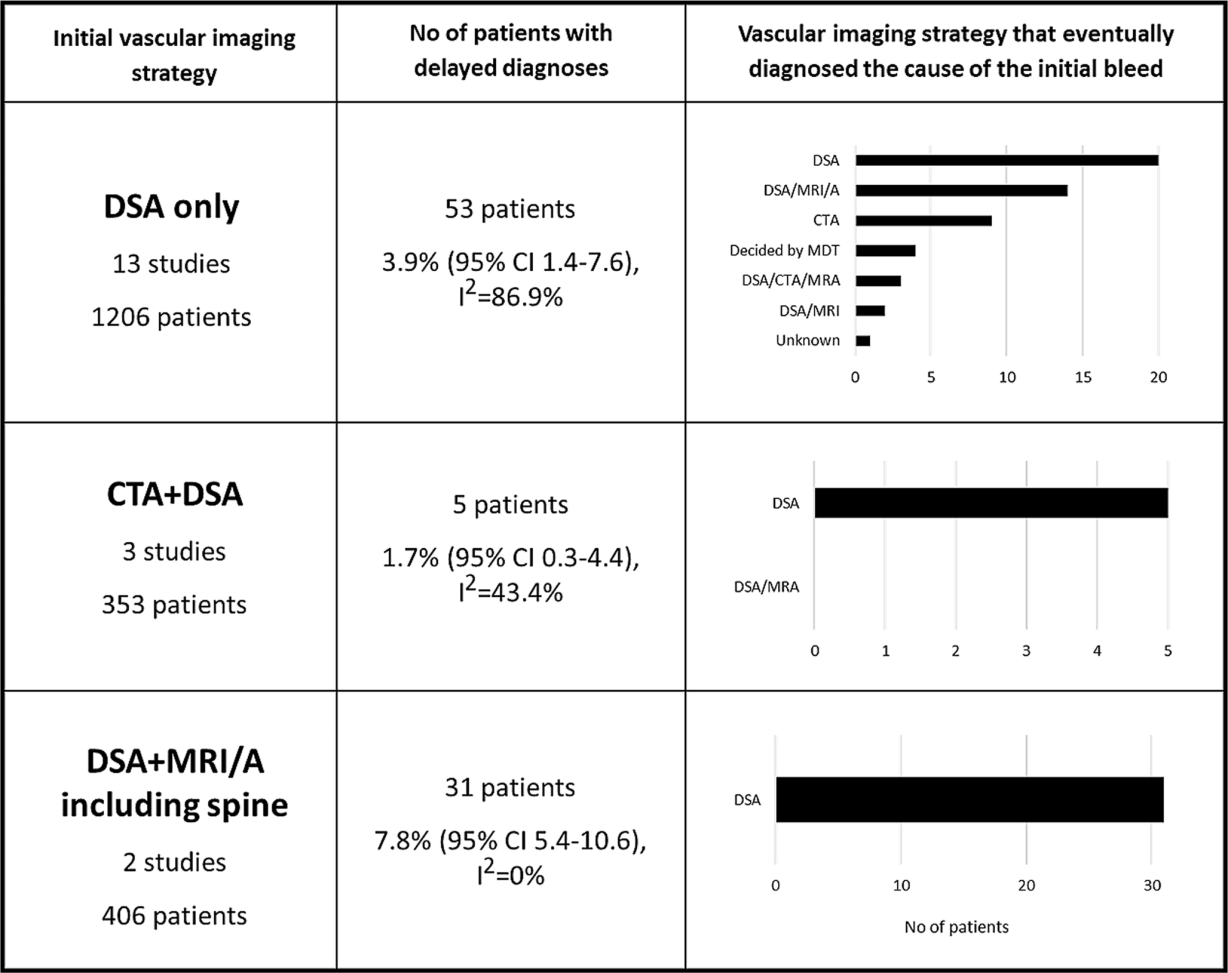
Risk of delayed diagnosis stratified by initial investigation strategy and details of imaging modalities by which delayed diagnoses were identified

The pooled rate of delayed diagnoses was 3.9% when DSA alone was the initial vascular imaging strategy, 1.7% when DSA and CTA were used and 7.8% when DSA and MRI/A (including spine) were used (Fig. 4). No studies reported delayed diagnoses data following CTA alone. The modality on which the delayed diagnoses were identified was DSA in most of the cases, although some were also identified on CTA (Fig. 4). Seven studies reported delayed diagnoses stratified by bleeding pattern: 3.5% [95% CI 1.0–7.5, *I*^2^ = 28.3%] for PnaSAH and 13.6% [95% CI 7.4–21.3, *I*^2^ = 74.3%] for non-PnaSAH. One study reported a single abnormality found on delayed imaging in 10 patients with radiologically negative SAH (10.0%).

### Functional outcomes

**Fig. 5:**
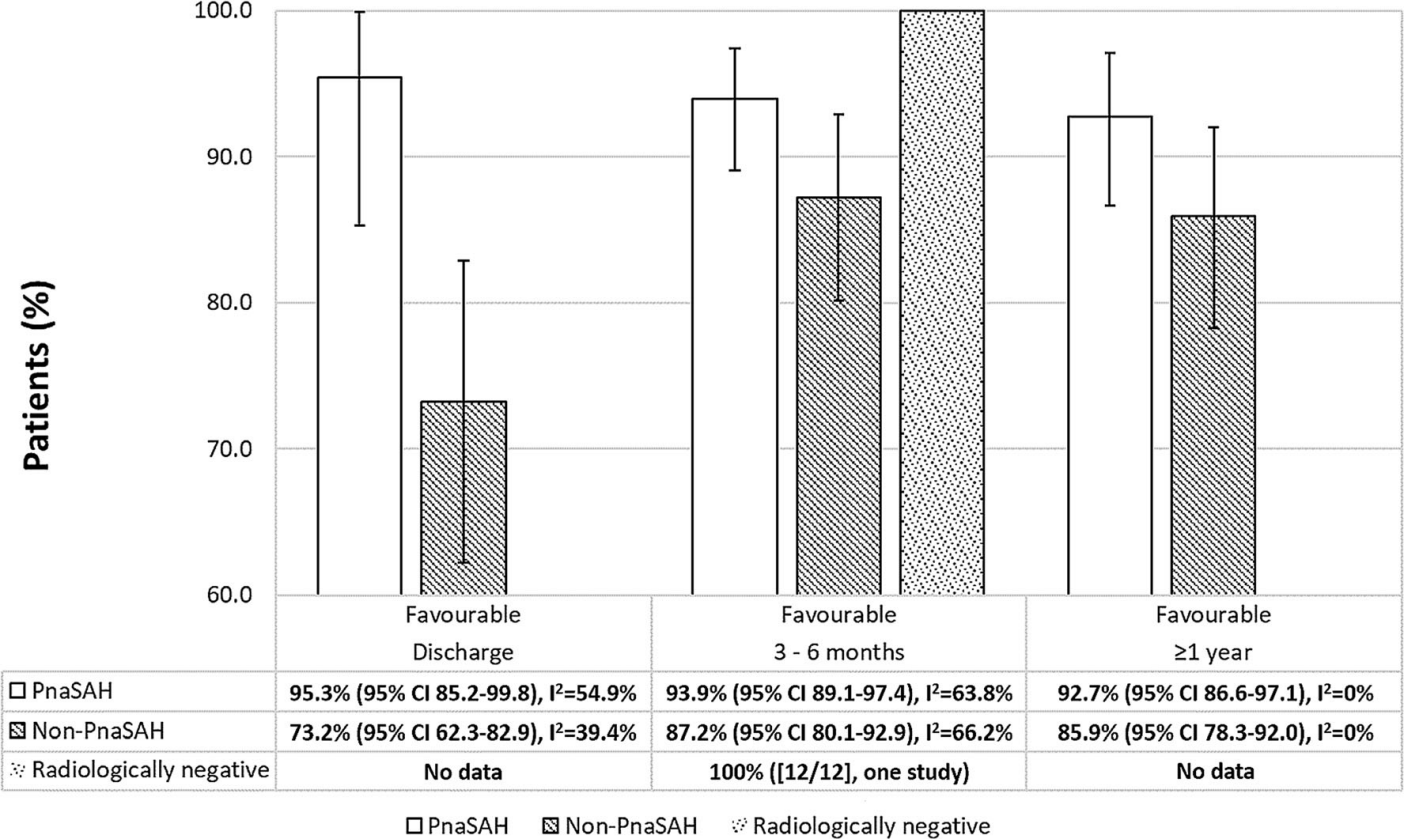
Modified Rankin scale outcomes stratified by bleeding pattern

Functional outcomes were reported in 17/58 studies (1402 patients). At discharge, outcomes were available for 273 patients (5 studies) and 177 had a favourable outcome (pooled proportion 86.5% [95% CI 80.3–91.6, *I*^2^ = 61.6%]). At 3–6 months, 685/774 patients (7 studies) had a favourable outcome (pooled proportion 92.0% [95% CI 86.0–96.5, *I*^2^ = 85.1%]). Two studies (190 patients) reported long-term outcomes (≥ 1 year); 171 had a favourable outcome (pooled proportion 89.6% [95% CI 86.6–97.1, *I*^2^ = 0%]). Outcomes stratified by bleeding pattern highlight that the likelihood of a poor outcome at 3–6 months and ≥ 1 year was double in non-PnaSAH patients when compared to PnaSAH patients (Fig. 5).

### Complications and length of hospital stay

Complications data were reported in 37/58 studies (3133 patients). Re-bleeding was reported in 23/58 studies (1675 patients) with a pooled risk of 3.1% [95% CI 1.5–5.2]. Hydrocephalus was reported in 28 studies (2399 patients) and the pooled risk was 16.0% [95% CI 11.2–21.4]. The pooled risk of vasospasm, reported in 19 studies (1926 patients), was 9.6% [95% CI 6.5–13.3]. Five studies (379 patients) reported incidence of seizures with a pooled risk of 3.5% [95% CI 1.7–5.8], although they did not specify whether these were early or late. The pooled risk of Na^+^ abnormalities, available in eight studies (547 patients), was 2.7% [95% CI 0.6–6.2]. Stratified by pattern of bleed, the risk of the above-mentioned complications in non-PnaSAH patients, except for Na^+^ abnormalities, was about double that of the PnaSAH cohort (Table 2).

**Table 2:** Post-bleed complications stratified by naSAH pattern of bleed

	All studies				Stratified by bleeding pattern									
	*N* of studies	Events/*N* of patients	% (95% CI)	*I*^2^, %	*N* of studies	PnaSAH, events/*N* ofpatients	% (95% CI)	*I*^2^, %	Non-PnaSAH, events/*N* of patients	% (95% CI)	*I*^2^, %	Radiologically negative, events/*N* of patients	% (95% CI)	*I*^2^, %
**Overall re-bleed**	23	56/1675	3.1 (1.5–5.2)	78.3	15	4/506	1.1 (0.4–2.2)	0	16/530	3.5 (1.8–5.8)	29.6	1/35	4.5 (0.3–13.6)	0
Early	17	45/1257	3.0 (1.1–5.9)	83.4	12	4/440	1.11 (0.4–2.3)	0	11/452	2.8 (1.1–5.4)	41.8	0/25	–	–
Late	9	11/824	1.6 (0.8–2.8)	20.4	6	0/213	0.5 (0.0–1.9)	0	5/244	2.6 (1.0–5.0)	2.4	1/35	4.5 (0.3–13.6)	0
**Overall hydrocephalus**	28	469/2399	16.0 (11.2–21.4)	91.3	23	104/915	9.0 (5.4–13.4)	76.5	241/924	25.5 (18.2–33.4)	85.4	0/39	1.6 (0.1–7.7)	0
Temporary	18	265/1571	16.3 (12.2–20.8)	80.1	15	59/541	10.2 (6.2–15.0)	62.1	122/660	21.8 (15.1–29.3)	78.3	0/39	1.6 (0.1–7.7)	0
Permanent	19	136/1711	7.2 (5.2–9.5)	64.4	15	24/704	3.5 (2.3–5.1)	4.8	79/628	13.0 (8.2–18.6)	71.7	–	–	–
**Overall vasospasm**	19	226/1926	9.6 (6.5–13.3)	83.3	14	62/706	6.9 (3.7–11.1)	70.6	144/803	13.5 (7.7–20.6)	85.6	1/103	1.8 (0.2–5.2)	0
DIND	4	19/463	4.4 (2.7–6.5)	5.8	3	0/106	0.7 (0.0–3.0)	0	6/110	6.4 (2.7–11.7)	0	0/14	3.1 (0.6–17.4)	0
Radiological	12	180/1435	11.3 (7.8–15.3)	78.1	11	49/530	8.3 (4.9–12.5)	55.2	116/630	14.0 (7.2–22.5)	86.1	1/99	1.7 (0.1–5.1)	0
**Seizures**	5	12/379	3.5 (1.7–5.8)	12.9	5	3/164	2.5 (0.7–5.5)	0	8/179	5.2 (2.4–8.9)	0	1/36	4.8 (0.4–13.9)	0
**Na** ^**+**^ **abnormalities**	8	17/547	2.7 (0.6–6.20	73.1	6	10/237	2.3 (0.0–7.9)	75.9	2/103	2.2 (0.0–7.6)	44.4	–	–	–

*DIND* delayed ischemic neurological deficit, *Na*^*+*^ sodium, *PnaSAH* perimesencephalic subarachnoid haemorrhage

Nine studies (608 patients) reported data regarding length of stay (Table 3) with the shortest median and mean for patients with PnaSAH being 10 and 8.3 days, respectively. For patients with non-PnaSAH, the shortest median and mean were 8.3 and 4 days, respectively. One study reported a median length of stay of 5 days for patients with radiologically negative SAH. The heterogeneity of formats in the reporting of length of stay means that meta-analysis could not be performed.

**Table 3:** Details regarding length of stay (days) for patients with naSAH stratified by pattern of bleed

Study	Overall, *N*	LOS	PnaSAH, *N*	LOS	Non-PnaSAH, *N*	LOS	Radiologically negative, *N*	LOS
Georgen et al., 1993	18	NR	9	12^a^	NR	NA	NR	NA
Andaluz et al., 2008	92	6.3^b^	45	4.3^b^	47	8.3^b^	0	NA
Beseoglu et al., 2010	21	15.3^b^	12	11.2^b^	9	20.7^b^	0	NA
Nayak et al., 2010	190	14^c^	NR	NA	NR	NA	NR	NA
Delgado Almandoz et al., 2012	25	2–7^a^	15	2–7^a^	8	2–7^a^	2	2–7
	34	8–14^a^	12	8–14^a^	20	8–14^a^	2	8–14
	13	≥ 15^a^	2	≥ 15^a^	11	≥ 15^a^	0	≥ 15
Boswell et al., 2013	31	NR	14	12.5^b^	16	13.2^b^	1	NR
Khan et al., 2013	50	NR	17^c^	8.3	23^c^	10	10^c^	5
Muehlschlegel et al., 2013	93	11^c^	36	NR	48	NR	9	NR
Canneti et al., 2015	41	21.1^b^	17	17^b^	24	24^b^	0	NA

*LOS* length of stay, *NA* not applicable, *NR* not reported

^a^Discharged within

^b^Mean

^c^Median

### Additional outcomes

No data were available in the included studies to inform two outcomes that were stated in our protocol, namely cost and patient perception of information provided by the clinical team.

## Discussion

This systematic review highlights the heterogeneity in presentation, functional outcomes and frequency of delayed diagnoses of naSAH patients, which, given its increasing incidence [37], emphasises a need to clarify optimal management strategies. Specifically, it is an important step in highlighting the need for tailored management that takes into consideration the higher rate of delayed diagnoses and complications in the non-PnaSAH cohort.

This review raises several interesting findings. The primary outcome of interest in our protocol was functional outcome at 6 months post-ictus as we sought to better understand outcomes in a group of patients who are generally thought to have “good” functional outcomes. The results suggest that non-PnaSAH patients had double the proportion of unfavourable outcomes at 3–6 months (12.8 vs 6.1%) and ≥ 1 year (14.4 vs 7.3%) when compared to PnaSAH patients and higher rate of complications including re-bleeding (3.5 vs 1.1%), hydrocephalus (25.5 vs 9.0%), vasospasm (13.5 vs 6.9%) and seizures (5.2 vs 2.5%); interestingly, the rates of sodium abnormalities were similar between the groups (2.2 vs 2.3%). This subgroup of patients may benefit from early transfer to a neurosurgical unit for the prevention and management of potential complications. This closely mirrors the findings of other publications, which suggest similar outcome and complication rates to patients with aneurysmal SAH [3]. There is evidence that, even in PnaSAH, patients may have cognitive or neuropsychogical impairment that precludes return to their premorbid functional state [50, 59]. Some of these patients may require specialised neurological rehabilitation and identification of the long-term neuropsychological, cognitive and quality-of-life outcomes is clearly warranted. Identification of these outcomes may also have implications for resource provision in terms of longer-term neurological rehabilitation.

It also shows that clinicians use diverse neurovascular investigation strategies before classifying patients as “non-aneurysmal”. A large proportion of studies utilised DSA as part of the first-line strategy and only two studies used CTA solely as the initial imaging tool, both of which did not report their rates of delayed diagnoses. Contemporary practice commonly involves CTA as a first-line investigation, which has been shown to have high specificity and sensitivity [77]. In addition, the potential risks of a DSA need to be considered but were not reported by any of the studies in this review. In an era of centralised neurosurgical and neuroradiological services and high quality CTA, it seems pertinent to evaluate the added benefits of early DSA (which requires transfer of patients to regional neurosciences centre) if the patient has a low-risk pattern of bleeding (i.e. PnaSAH) and the initial CTA is of sufficient quality and does not identify a vascular abnormality [5]. The studies included in this review did not permit such an assessment. It is interesting, however, to note that the delayed pick-up rate of vascular abnormalities in patients who had had a CTA and DSA as their initial strategy was as low as 1.7%.

Another poorly understood entity in acute SAH, especially in the context of a negative CTA, is the role of early MR imaging, including MRA and spinal imaging. Interestingly, our study identified a high delayed diagnosis rate of 7.8% in patients who had had initial DSA and MRI/A and the reasons behind this warrant further exploration. A recent study showed high sensitivities (98.2%) but slightly lower specificities (91%) of 3.0 T MRA in detecting vascular abnormalities in a large cohort of acute good-grade SAH patients, indicating that it could be a useful tool even in the acute setting [45].

To our knowledge, there are no published guidelines as to which imaging modalities to use for repeat imaging and the timeframe for when this repeat imaging should be undertaken. We found that delayed diagnoses were almost 4× more prevalent in the non-PnaSAH group compared to the PnaSAH group and so it would seem logical to investigate patients with non-PnaSAH more comprehensively and as early as possible to facilitate early treatment of vascular abnormalities, avoid re-bleeding events and thereby optimise functional outcomes. Other risk factors for delayed diagnosis, that warrant early aggressive investigation, also require characterisation. It is interesting to note that the majority of delayed diagnoses were identified on follow-up DSA, although this may simply be a consequence of DSA being the predominant investigation used in the delayed setting; the yield of non-invasive techniques in this delayed setting also requires evaluation. For the time-being, and in view of the marked heterogeneity of initial investigation regimens, and the poor reporting of timing of delayed imaging and diagnoses, specific recommendations regarding follow-up imaging cannot be made and tailored to each bleeding pattern.

These data make a strong case for a large-scale multi-centre prospective observational studies to better understand the nuances of delayed diagnoses, functional outcomes and complications in patients with naSAH. Studying a large consecutive prospective cohort with individual patient-level data will allow inferences to be made about the utility of various imaging modalities, particularly if Bayesian statistical models are incorporated, which allow assessments of probabilities of specified outcomes (functional outcome, complication, delayed diagnosis) based on priors. This will then enable us to formulate nuanced individualised strategies that optimise patient outcomes allowing stratification of patients that may require early and/or delayed angiography, or management of acute or chronic neurological complications. Furthermore, economic analyses need to be undertaken to specifically address the issue of patient transfers to specialist neuroscience units for further investigation and management, an aspect that was not reported in any of the included studies.

Perhaps an under-appreciated aspect is the patient perspective; these patients are often left with a sense of uncertainty surrounding their prognosis and need for further investigation and interventions [65], although these were not reported in any of the studies included in this review. Therefore, studying what information is conveyed to the patient also seems prudent; specifically, in an era of shared decision making, the presentation of the risk-benefit balance and the involvement of the patient and family in the decision-making process becomes all the more important.

Some limitations of this study should be noted. Most studies included were single-centre retrospective studies, limited by inherent biases. This reflected in the highly heterogeneous meta-analyses and quality assessment results, and as such, caution should be exercised in interpretation of pooled proportions. However, given that the majority of the studies in this field are retrospective, of which some were assessed in our quality assessment tool as “good”, we chose to continue to include these in the systematic review and meta-analysis. Especially in a field with high heterogeneity, we found evidence that including multiple study designs is warranted [64]. Furthermore, radiological breakdown of non-PnaSAH into cortical or basal cistern patterns was not feasible with the data available. Timing between ictus and CT was not extracted. SAH blood normally resorbs over time, and a scan at 72 h suggesting a PnaSAH may well represent the residual blood from a non-PnaSAH. The data is also historical, with many of the studies using DSA as the initial imaging modality; it would be prudent to assess the prevalence of naSAH and delayed diagnosis rates in an era of widespread use of high-resolution CTA. It was not within the scope of this review to determine the diagnostic accuracy of the different imaging modes of a diagnostic modality and as such data regarding resolution among other imaging parameters were not extracted. Risk of complications of CTA, DSA and MRI/A was not determined based on data available in our selected studies; however, this is an important aspect to consider against the risk of delayed diagnosis in naSAH patients and particularly those with a perimesencephalic pattern.

### Heterogeneity and external validity

The strengths of this study with regard to external validity include the large pooled population from diverse geographical locations, the primarily uniform definitions to describe groups of bleeding pattern and the agreement with previous studies on the association between bleeding pattern and delayed diagnoses outcomes [33, 56, 62]. However, the reliability of our results is dependent on the quality of data available in the included studies, and selection and recall bias may influence the generalisability of our findings; data was pooled from populations with differing and often unknown baseline characteristics, for example, data regarding use of antiplatelet and anticoagulant drugs were not available. Furthermore, possible interactions among variables associated with post-bleed complications and functional outcomes, such as clinico-radiological severity and bleeding pattern, could not be accounted for as the nature of the data available precluded multivariate or individual patient-level analyses. Significant variation in outcome measures was also observed. All of these are reflected in the large *I*^2^ values in many of the meta-analyses, and therefore, caution should be exercised in interpreting some of these results. These limitations and high *I*^2^ statistics highlight the fact that there is significant heterogeneity within the pre-defined subgroups and suggest that decisions about management and further investigation require large volumes of individual patient-level data from carefully curated prospective databases to understand the nuances of how to tailor management to detect occult vascular causes and optimise functional outcomes. There is also a clear need to report studies according to the newly developed set of common data elements and develop a patient-centred core outcome set for data standardisation to allow future homogenous analyses that will benefit both patients and clinicians [60].

## Conclusions

There is much heterogeneity in how patients with spontaneous SAH and negative initial angiographic imaging are investigated and treated. Although there are a higher proportion of patients with favourable functional outcome in the PnaSAH cohort compared to the non-PnaSAH cohort, both cohorts have patients (6.1 and 12.8%, respectively) with unfavourable functional outcomes that may be optimised with better acute management of complications and longer-term rehabilitation. There is a higher rate of delayed diagnoses in the non-PnaSAH cohort. However, with the heterogeneity of initial and delayed neurovascular imaging strategies, it is challenging to attribute the delayed discovery to a single patient aspect, be it clinical or radiological. This systematic review therefore highlights the need for an evidence-based, stratified approach to this patient cohort based upon the initial pattern of bleeding and clinico-radiological status. There is a pressing need for a contemporary large-scale prospective multi-centre observational study that would facilitate the identification of high-risk patients that require comprehensive investigation and management in neuroscience centres with specific neurorehabilitation requirements and potentially low-risk patients that can be managed with more conservative strategies.

## Electronic supplementary material

ESM 1 (DOCX 632 kb)
