# Supplementary material for: Subarachnoid haemorrhage with negative initial neurovascular imaging: a systematic review and meta-analysis
Source: Acta Neurochir (Wien). 2019 Aug 13;161(10):2013–26. doi: 10.1007/s00701-019-04025-w (PMC6739283; doi:10.1007/s00701-019-04025-w)
Supplement: Supplementary file 1 — (DOCX 632 kb) [file 701_2019_4025_MOESM1_ESM.docx]

# **SUPPLEMENTARY MATERIAL AND REFERENCES**

SUBARACHNOID HAEMORRHAGE WITH NEGATIVE INITIAL NEUROVASCULAR IMAGING: A SYSTEMATIC REVIEW AND META-ANALYSIS

Midhun Mohan, MRes, Abdurrahman I. Islim, MPhil, Fahid T. Rasul, FRCS, Ola Rominiyi, MRCS, Ruth-Mary deSouza, MRCS, Michael T.C. Poon, MSc, Aimun A.B. Jamjoom, PhD, Angelos G. Kolias, PhD, Julie Woodfield, MSc, Krunal Patel, FRCS, Aswin Chari, MRCS, Ramez Kirollos, MD, on behalf of the British Neurosurgical Trainee Research Collaborative

| **Table e-1. Study characteristics** | | | | | |
| --- | --- | --- | --- | --- | --- |
| Authors | Year | Study | N. of Patients | Mean age (years) | Male (%) |
| Rinkel et al. [48] | 1991 | Retrospective | 65 | 53 | 61.50% |
| Goergen et al. [19] | 1993 | Retrospective | 18 | NR | NR |
| Van Calenberghet al. [53] | 1993 | Retrospective | 62 | 47 | 40.00% |
| Hutter et al. [21] | 1994 | Retrospective | 20 | 49 | NR |
| Canhao et al. [9] | 1995 | Prospective and retrospective | 71 | 49.9 | 56.00% |
| Tatter et al. [50] | 1995 | Retrospective | 40 | NR | NR |
| Duong et al. [15] | 1996 | Prospective | 92 | 49 | 54.00% |
| Berdoz et al. [4] | 1998 | Retrospective | 52 | 52.8 | 65.00% |
| Linn et al. [35] | 1998 | Prospective | 23 | 56 | 74.00% |
| Madureira et al. [36] | 2000 | prospective | 18 | NR | NR |
| Marquardt et al. [37] | 2000 | Prospective | 21 | 55 | 52.00% |
| Franz et al. [18] | 2001 | Retrospective | 34 | 50.09 | 52.00% |
| Ildan et al. [22] | 2002 | Retrospective | 84 | 49.5 | NR |
| Alen et al. [1] | 2003 | Prospective | 44 | 51.9 | 63.60% |
| Lang et al. [33] | 2003 | Prospective | 57 | 54.68 | 56.00% |
| Topcuoglu et al. [51] | 2003 | Retrospective | 86 | 54.3 | 63.00% |
| Caeiro et al. [7] | 2005 | Prospective | 33 | NR | NR |
| Jung et al. [23] | 2006 | Prospective | 143 | 52.3 | 42.00% |
| Matsuyama et al. [39] | 2006 | Prospective | 9 | 50 | 57.00% |
| Andaluz et al. [3] | 2008 | Retrospective | 92 | 49.4 | 34.00% |
| Kang et al. [24] | 2009 | Retrospective | 52 | 55.4 | 53.80% |
| Whiting et al. [55] | 2009 | Retrospective | 89 | 56 | 51.00% |
| Beseoglu et al. [5] | 2010 | Retrospective | 21 | 57.19 | 60.00% |
| Caeiro et al. [8] | 2010 | Prospective | 37 | NR | 43.00% |
| Nayak et al. [43] | 2010 | Retrospective | 190 | 57 | 61.60% |
| Alfieri et al. [2] | 2011 | Prospective | 38 | 44.3 | NR |
| Fontanella et al. [17] | 2011 | Retrospective | 102 | 53 | 62.70% |
| Kong et al. [30] | 2011 | Prospective | 31 | 49.7 | NR |
| Oda et al. [44] | 2011 | Retrospective | 15 | NR | NR |
| Pyysalo et al. [46] | 2011 | Retrospective | 97 | 52 | 36.00% |
| Cánovas et al. [11] | 2012 | Retrospective | 108 | 52.4 | 44.90% |
| Delgado Almandoz et al. [14] | 2012 | Retrospective | 72 | 53.1 | 36.10% |
| Gross et al. [20] | 2012 | Retrospective | 77 | 59.8 | 48.00% |
| Kostic et al. [31] | 2012 | Prospective | 36 | 48.3 | 55.60% |
| Lin et al. [34] | 2012 | Prospective | 68 | 59.5 | 51.50% |
| Maslehaty et al. [38] | 2012 | Prospective and retrospective | 179 | NR | 60.00% |
| Yu et al. [57] | 2012 | Retrospective | 28 | 60.3 | 51.10% |
| Zhong et al. [58] | 2012 | Retrospective | 49 | 54.04 | 49.00% |
| Boswell et al. [6] | 2013 | Retrospective | 31 | 56.3 | 55.00% |
| Dalyai et al. [13] | 2013 | Retrospective | 254 | NR | NR |
| Khan et al. [25] | 2013 | Retrospective | 50 | 52.5 | 60.00% |
| Muehlschlegel et al. [42] | 2013 | Retrospective | 93 | 54 | 59.00% |
| Pratet al. [45] | 2013 | Retrospective | 63 | 52.4 | 54.00% |
| Tsermoulas et al. [52] | 2013 | Retrospective | 62 | 51 | 51.20% |
| Woodfield et al. [56] | 2013 | Retrospective | 240 | 51 | 62.00% |
| Ellis et al. [16] | 2014 | Prospective | 173 | 55 | 55.00% |
| Konczalla et al. [27] | 2014 | Prospective | 125 | 56 | 70.00% |
| Kumar et al. [32] | 2014 | Prospective | 39 | 50.5 | 69.00% |
| Mensing [40] et al. | 2014 | Prospective | 79 | 53 | 54.00% |
| Qureshi et al. [47] | 2014 | Retrospective | 5 | 59.8 | NR |
| Canneti et al. [10] | 2015 | Retrospective | 41 | 54.98 | 51.00% |
| Dalbjerg et al. [12] | 2015 | Retrospective | 95 | 53 | 48.40% |
| Konczalla et al. [29] | 2015 | Retrospective | 152 | 58 | 59.00% |
| Konczalla et al. [28] | 2015 | Prospective | 173 | 56 | NR |
| Sprenker et al. [49] | 2015 | Retrospective | 26 | NR | NR |
| Walcott et al. [54] | 2015 | Retrospective | 138 | 55.6 | 53.60% |
| Konczalla et al. [26] | 2016 | Retrospective | 225 | 57 | NR |
| Moscovici et al. [41] | 2016 | Retrospective | 56 | 53.4 | 54.00% |

| **Table e-2. Delayed diagnoses in 18 studies that reported this outcome** | | | | | | | |
| --- | --- | --- | --- | --- | --- | --- | --- |
| Authors | Year | N. of patients | Delayed diagnoses | (%) | Initial Neurovascular Imaging | Follow-up imaging modality that diagnosed the vascular abnormality | Time frame of follow-up imaging |
| Rinkel et al. [48] | 1991 | 65 | 0 | 0.00% | DSA | Further DSA | NR |
| Hütter et al. [21] | 1994 | 20 | 0 | 0.00% | DSA | Unknown | NR |
| Berdoz et al. [4] | 1998 | 52 | 1 | 1.90% | DSA | Unknown | NR |
| Topcuoglu et al. [51] | 2003 | 86 | 3 | 3.50% | DSA | Further DSA in most (rest had CTA/MRA) | NR |
| Jung et al. [23] | 2006 | 143 | 18 | 12.60% | DSA | Further DSA | NR |
| Kang et al. [24] | 2009 | 52 | 9 | 17.30% | DSA | CTA | At least 1 year after |
| Pyysalo et al. [46] | 2011 | 97 | 0 | 0.00% | DSA | Further DSA (some also had MRI) | NR |
| Maslehaty et al. [38] | 2012 | 179 | 14 | 7.80% | DSA | MRI/A brain and C-spine within 72h (most also had further DSA) | Within 72 hours |
| Delgado Almandoz et al. [14] | 2012 | 72 | 3 | 4.20% | DSA+CTA | Further DSAs | 1 and 12 weeks |
| Kostic et al. [31] | 2012 | 36 | 2 | 5.60% | DSA | Further DSA (some also had MRI) | NR |
| Yu et al. [57] | 2012 | 28 | 2 | 7.10% | DSA | Further DSA | NR |
| Dalyai et al. [13] | 2013 | 254 | 17 | 6.70% | DSA+MRI/A brain +MRI C-spine | Further DSAs | 1 and 6 weeks |
| Khan et al. [25] | 2013 | 50 | 4 | 8.00% | DSA | Individualised, decided by MDT | NR |
| Woodfield et al. [56] | 2014 | 240 | 2 | 0.80% | DSA+CTA | Further DSA | NR |
| Konczalla et al. [29] | 2014 | 152 | 14 | 9.20% | DSA+Spinal MRI | Further DSA | NR |
| Konczalla et al. [28] | 2015 | 173 | 0 | 0.00% | DSA | Further DSAs (some also had MRI head and spine) | NR |
| Canneti et al. [10] | 2015 | 41 | 0 | 0.00% | DSA+CTA | Further DSA in most (rest had MRA) | NR |
| Konczalla et al. [26] | 2016 | 225 | 0 | 0.00% | DSA | Further DSA | NR |
| Abbreviations: CTA=computed tomography angiography; DSA=digital subtraction angiography; MRI/A=magnetic resonance imaging/angiography; NR=not reported | | | | | | | |


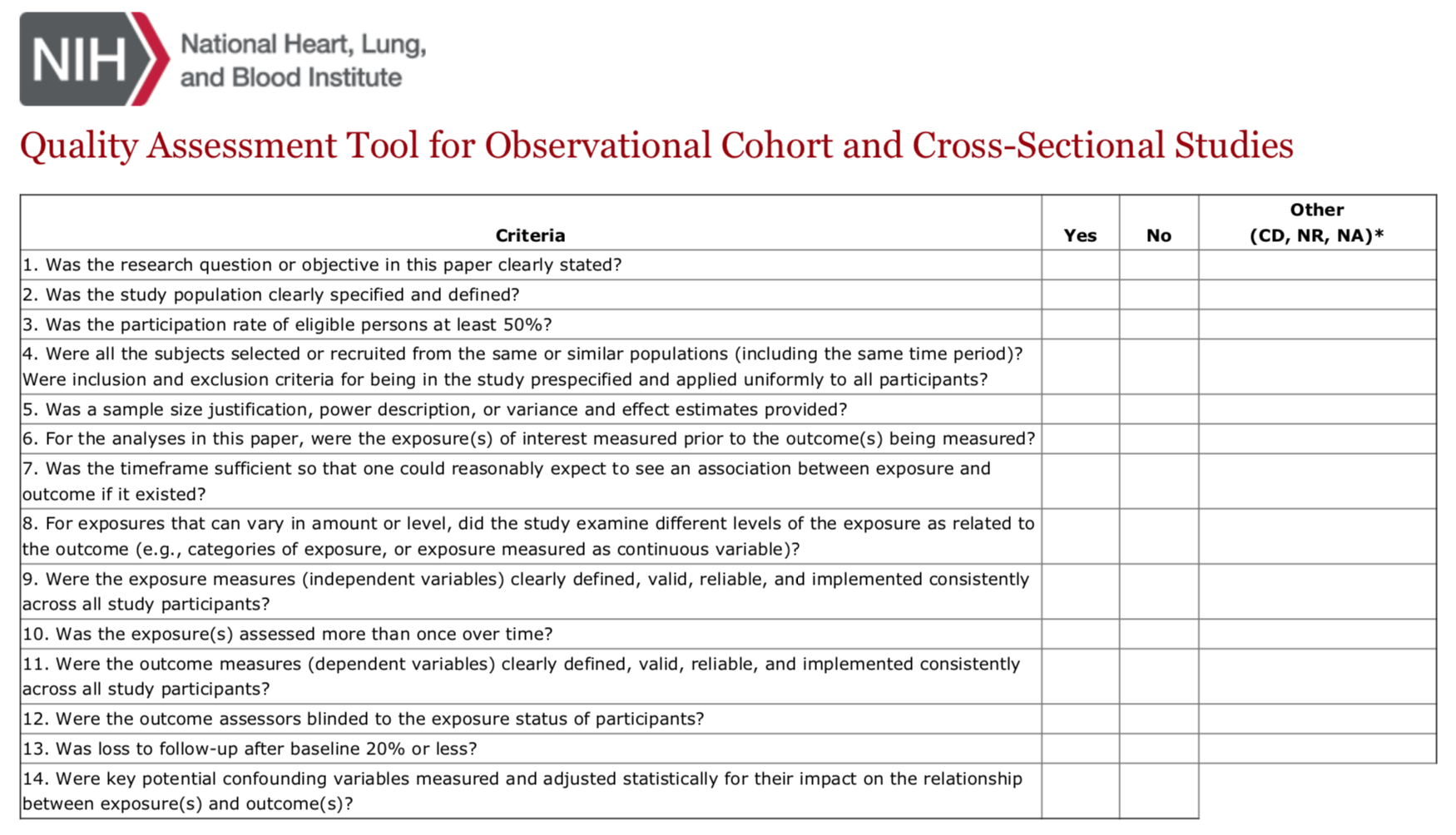


**Figure e-1. NIH Quality Assessment Tool for Observational Cohort and Cross-Sectional Studies**

**
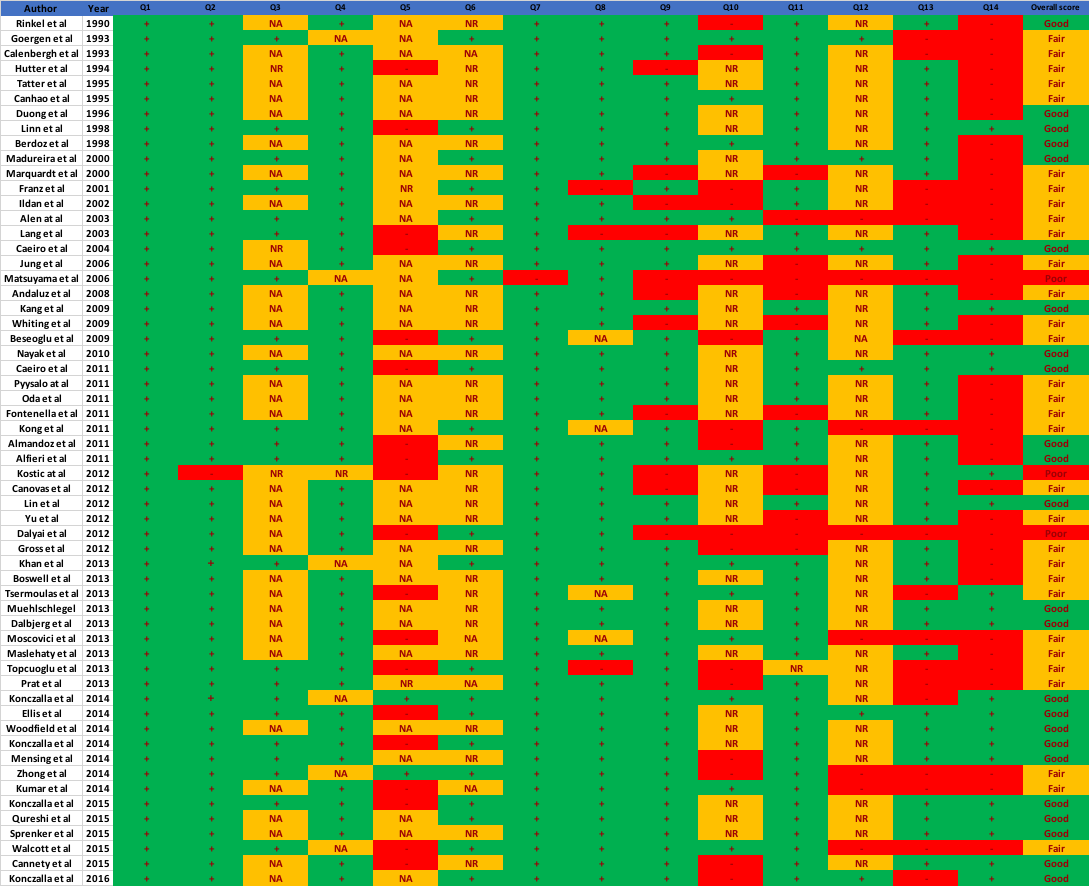
**

**Figure e-2. Quality assessment results**

# **SUPPLEMENTARY REFERENCES**

1. Alén JF, Lagares A, Lobato RD, Gómez PA, Rivas JJ, Ramos A (2003) Comparison between perimesencephalic nonaneurysmal subarachnoid hemorrhage and subarachnoid hemorrhage caused by posterior circulation aneurysms. Journal of neurosurgery 98:529-535. doi:10.3171/jns.2003.98.3.0529

2. Alfieri A, Gazzeri R, Pircher M, Unterhuber V, Schwarz A (2011) A prospective long-term study of return to work after nontraumatic nonaneurysmal subarachnoid hemorrhage. Journal of clinical neuroscience : official journal of the Neurosurgical Society of Australasia 18:1478-1480

3. Andaluz N, Zuccarello M (2008) Yield of further diagnostic work-up of cryptogenic subarachnoid hemorrhage based on bleeding patterns on computed tomographic scans. Neurosurgery 62:1040-1046- discussion 1047. doi:10.1227/01.neu.0000325865.22011.1f

4. Berdoz D, Uske A, De Tribolet N (1998) Subarachnoid haemorrhage of unknown cause: Clinical, neuroradiological and evolutive aspects. Journal of clinical neuroscience : official journal of the Neurosurgical Society of Australasia 5:274-282

5. Beseoglu K, Pannes S, Steiger HJ, Hänggi D (2009) Long-term outcome and quality of life after nonaneurysmal subarachnoid hemorrhage. Acta neurochirurgica 152:409-416

6. Boswell S, Thorell W, Gogela S, Lyden E, Surdell D (2013) Angiogram-Negative Subarachnoid Hemorrhage: Outcomes Data and Review of the Literature. Journal of Stroke and Cerebrovascular Diseases 22:750-757

7. Caeiro L, Menger C, Ferro JM, Albuquerque R, Figueira ML (2005) Delirium in Acute Subarachnoid Haemorrhage. Cerebrovascular Diseases 19:31-38. doi:10.1159/000081909

8. Caeiro L, Santos CO, Ferro JM, Figueira ML (2010) Neuropsychiatric disturbances in acute subarachnoid haemorrhage. European Journal of Neurology 18:857-864

9. Canhão P, Ferro JM, Pinto AN, Melo TP, Campos JG (1995) Perimesencephalic and nonperimesencephalic subarachnoid haemorrhages with negative angiograms. Acta neurochirurgica 132:14-19

10. Canneti B, Mosqueira AJ, Nombela F, Gilo F, Vivancos J (2015) Spontaneous Subarachnoid Hemorrhage with Negative Angiography Managed in a Stroke Unit: Clinical and Prognostic Characteristics. Journal of Stroke & Cerebrovascular Diseases 24:2484-2490. doi:10.1016/j.jstrokecerebrovasdis.2015.06.011

11. Cánovas D, Gil A, Jato M, de Miquel M, Rubio F (2011) Clinical outcome of spontaneous non-aneurysmal subarachnoid hemorrhage in 108 patients. European Journal of Neurology 19:457-461

12. Dalbjerg SM, Larsen CC, Romner B (2013) Risk factors and short-term outcome in patients with angiographically negative subarachnoid hemorrhage. Clinical neurology and neurosurgery 115:1304-1307

13. Dalyai R, Chalouhi N, Theofanis T, Jabbour PM, Dumont AS, Gonzalez LF, Gordon DS, Thakkar V, Rosenwasser RH, Tjoumakaris SI (2013) Subarachnoid hemorrhage with negative initial catheter angiography: a review of 254 cases evaluating patient clinical outcome and efficacy of short- and long-term repeat angiography. Neurosurgery 72:646-652- discussion 651-642. doi:10.1227/NEU.0b013e3182846de8

14. Delgado Almandoz JE, Jagadeesan BD, Refai D, Moran CJ, Cross DT, 3rd, Chicoine MR, Rich KM, Diringer MN, Dacey RG, Jr., Derdeyn CP, Zipfel GJ (2012) Diagnostic yield of computed tomography angiography and magnetic resonance angiography in patients with catheter angiography-negative subarachnoid hemorrhage. J Neurosurg 117:309-315. doi:10.3171/2012.4.jns112306

15. Duong H, Melançon D, Tampieri D, Ethier R (1996) The negative angiogram in subarachnoid haemorrhage. Neuroradiology 38:15-19

16. Ellis JA, McDowell MM, Mayer SA, Lavine SD, Meyers PM, Connolly Jr ES (2014) The Role of Antiplatelet Medications in Angiogram-Negative Subarachnoid Hemorrhage. Neurosurgery 75:530-535

17. Fontanella M, Rainero I, Panciani PP, Schatlo B, Benevello C, Garbossa D, Carlino C, Valfrè W, Griva F, Bradac GB, Ducati A (2011) Subarachnoid hemorrhage and negative angiography: clinical course and long-term follow-up. Neurosurgical review 34:477-484

18. Franz G, Brenneis C, Kampfl A, Pfausler B, Poewe W, Schmutzhard E (2001) Prognostic value of intraventricular blood in perimesencephalic nonaneurysmal subarachnoid hemorrhage. Journal of computer assisted tomography 25:742-746

19. Goergen SK, Barrie D, Sacharias N, Waugh JR (1993) Perimesencephalic subarachnoid haemorrhage: negative angiography and favourable prognosis. Australasian radiology 37:156-160

20. Gross BA, Lin N, Frerichs KU, Du R (2012) Vasospasm after spontaneous angiographically negative subarachnoid hemorrhage. Acta neurochirurgica 154:1127-1133

21. Hütter BO, Gilsbach JM, Kreitschmann I (1994) Is there a difference in cognitive deficits after aneurysmal subarachnoid haemorrhage and subarachnoid haemorrhage of unknown origin? Acta neurochirurgica 127:129-135

22. Ildan F, Tuna M, Erman T, Göçer AI, Cetinalp E (2002) Prognosis and prognostic factors in nonaneurysmal perimesencephalic hemorrhage: a follow-up study in 29 patients. Surgical neurology 57:160-165- discussion 165-166

23. Jung JY, Kim YB, Lee JW, Huh SK, Lee KC (2006) Spontaneous subarachnoid haemorrhage with negative initial angiography: A review of 143 cases. Journal of Clinical Neuroscience 13:1011-1017

24. Kang DH, Park J, Lee SH, Park SH, Kim YS, Hamm IS (2009) Does non-perimesencephalic type non-aneurysmal subarachnoid hemorrhage have a benign prognosis? Journal of Clinical Neuroscience 16:904-908. doi:<http://dx.doi.org/10.1016/j.jocn.2008.10.008>

25. Khan AA, Smith JDS, Kirkman MA, Robertson FJ, Wong K, Dott C, Grieve JP, Watkins LD, Kitchen ND (2013) Angiogram negative subarachnoid haemorrhage: Outcomes and the role of repeat angiography. Clinical neurology and neurosurgery 115:1470-1475

26. Konczalla J, Kashefiolasl S, Brawanski N, Lescher S, Senft C, Platz J, Seifert V (2016) Cerebral vasospasm and delayed cerebral infarctions in 225 patients with non-aneurysmal subarachnoid hemorrhage: the underestimated risk of Fisher 3 blood distribution. Journal of NeuroInterventional Surgery 8:1247-1252

27. Konczalla J, Platz J, Schuss P, Vatter H, Seifert V, Guresir E (2014) Non-aneurysmal non-traumatic subarachnoid hemorrhage: Patient characteristics, clinical outcome and prognostic factors based on a single-center experience in 125 patients. BMC Neurology 14:140. doi:<http://dx.doi.org/10.1186/1471-2377-14-140>

28. Konczalla J, Schmitz J, Kashefiolasl S, Senft C, Seifert V, Platz J (2015) Non-aneurysmal subarachnoid hemorrhage in 173 patients: a prospective study of long-term outcome. European Journal of Neurology 22:1329-1336

29. Konczalla J, Schuss P, Platz J, Vatter H, Seifert V, Güresir E (2014) Clinical outcome and prognostic factors of patients with angiogram-negative and non-perimesencephalic subarachnoid hemorrhage: benign prognosis like perimesencephalic SAH or same risk as aneurysmal SAH? Neurosurgical review 38:121-127. doi:10.1007/s10143-014-0568-0

30. Kong Y, Zhang JH, Qin X (2011) Perimesencephalic Subarachnoid Hemorrhage: Risk Factors, Clinical Presentations, and Outcome. In., vol Chapter 34. Springer Vienna, Vienna, pp 197-201. doi:10.1007/978-3-7091-0353-1_34

31. Kostic A, Stojanov D, Stefanovic I, Novak V, Kostic E, Benedeto-Stojanov D, Veselinovic D (2012) Complications after angiogram-negative subarachnoid haemorrhage: Comparative study of pretruncal and nonpretruncal hemorrhage patients. Srpski arhiv za celokupno lekarstvo 140:8-13

32. Kumar R, Das KK, Sahu RK, Sharma P, Mehrotra A, Srivastava AK, Sahu RN, Jaiswal AK, Behari S (2014) Angio negative spontaneous subarachnoid hemorrhage: Is repeat angiogram required in all cases? Surg Neurol Int 5. doi:10.4103/2152-7806.138367

33. Lang EW, Khodair A, Barth H, Hempelmann RG, Dorsch NWC, Mehdorn HM (2003) Subarachnoid hemorrhage of unknown origin and the basilar artery configuration. Journal of Clinical Neuroscience 10:74-78. doi:<http://dx.doi.org/10.1016/S0967-5868%2802%2900124-8>

34. Lin N, Zenonos G, Kim AH, Nalbach SV, Du R, Frerichs KU, Friedlander RM, Gormley WB (2012) Angiogram-Negative Subarachnoid Hemorrhage: Relationship Between Bleeding Pattern and Clinical Outcome. Neurocritical care 16:389-398

35. Linn FH, Rinkel GJ, Algra A, van Gijn J (1998) Headache characteristics in subarachnoid haemorrhage and benign thunderclap headache. Journal of Neurology, Neurosurgery & Psychiatry 65:791-793

36. Madureira S, Canhao P, Guerreiro M, Ferro JM (2000) Cognitive and emotional consequences of perimesencephalic subarachnoid hemorrhage. J Neurol 247:862-867

37. Marquardt G, Niebauer T, Schick U, Lorenz R (2000) Long term follow up after perimesencephalic subarachnoid haemorrhage. Journal of neurology, neurosurgery, and psychiatry 69:127-130

38. Maslehaty H, Petridis AK, Barth H, Mehdorn HM (2011) Diagnostic value of magnetic resonance imaging in perimesencephalic and nonperimesencephalic subarachnoid hemorrhage of unknown origin: Clinical article. Journal of Neurosurgery 114:1003-1007. doi:<http://dx.doi.org/10.3171/2010.6.JNS10310>

39. Matsuyama T, Okuchi K, Seki T, Higuchi T, Murao Y (2006) Perimesencephalic nonaneurysmal subarachnoid hemorrhage caused by physical exertion. Neurologia medico-chirurgica 46:277-281- discussion 281-272

40. Mensing LA, Ruigrok YM, Greebe P, Vlak MHM, Algra A, Rinkel GJE (2014) Risk factors in patients with perimesencephalic hemorrhage. European Journal of Neurology 21:816-819

41. Moscovici S, Fraifeld S, Ramirez-de-Noriega F, Rosenthal G, Leker RR, Itshayek E, Cohen JE (2013) Clinical relevance of negative initial angiogram in spontaneous subarachnoid hemorrhage. Neurological Research 35:117-122. doi:<http://dx.doi.org/10.1179/1743132812Y.0000000147>

42. Muehlschlegel S, Kursun O, Topcuoglu MA, Fok J, Singhal AB (2013) Differentiating Reversible Cerebral Vasoconstriction Syndrome With Subarachnoid Hemorrhage From Other Causes of Subarachnoid Hemorrhage. JAMA Neurology:1-7. doi:10.1001/jamaneurol.2013.3484

43. Nayak S, Kunz AB, Kieslinger K, Ladurner G, Killer M, Nayak S, Kunz AB, Kieslinger K, Ladurner G, Killer M (2010) Classification of non-aneurysmal subarachnoid haemorrhage: CT correlation to the clinical outcome. Clinical Radiology 65:623-628. doi:10.1016/j.crad.2010.01.022

44. Oda S, Shimoda M, Hoshikawa K, Osada T, Yoshiyama M, Matsumae M (2011) Cortical subarachnoid hemorrhage caused by cerebral venous thrombosis. Neurologia medico-chirurgica 51:30-36

45. Prat D, Goren O, Bruk B, Bakon M, Hadani M, Harnof S (2013) Description of the Vasospasm Phenomena following Perimesencephalic Nonaneurysmal Subarachnoid Hemorrhage. BioMed Research International 2013:1-8

46. Pyysalo LM, Niskakangas TT, Keski-Nisula LH, Kahara VJ, Ohman JE (2011) Long term outcome after subarachnoid haemorrhage of unknown aetiology. Journal of Neurology, Neurosurgery & Psychiatry 82:1264-1266

47. Qureshi AI, Jahangir N, Qureshi MH, Defillo A, Malik AA, Sherr GT, Suri MFK (2014) A Population-Based Study of the Incidence and Case Fatality of Non-aneurysmal Subarachnoid Hemorrhage. Neurocritical care 22:409-413

48. Rinkel GJ, Wijdicks EF, Vermeulen M, Hasan D, Brouwers PJ, van Gijn J (1991) The clinical course of perimesencephalic nonaneurysmal subarachnoid hemorrhage. Annals of neurology 29:463-468. doi:10.1002/ana.410290503

49. Sprenker C, Jaymin P, Camporesi E, Vasan R, Van Loveren H, Chen H, Agazzi S (2015) Medical and neurologic complications of the current management strategy of angiographically negative nontraumatic subarachnoid hemorrhage patients. Journal of critical care 30:216.e217-216.e211

50. Tatter SB, Crowell RM, Ogilvy CS (1995) Aneurysmal and Microaneurysmal “Angiogram-negative” Subarachnoid Hemorrhage. Neurosurgery 37:48-55. doi:10.1227/00006123-199507000-00007

51. Topcuoglu MA, Ogilvy CS, Carter BS, Buonanno FS, Koroshetz WJ, Singhal AB (2003) Subarachnoid hemorrhage without evident cause on initial angiography studies: diagnostic yield of subsequent angiography and other neuroimaging tests. Journal of neurosurgery 98:1235-1240

52. Tsermoulas G, Flett L, Gregson B, Mitchell P (2013) Immediate coma and poor outcome in subarachnoid haemorrhage are independently associated with an aneurysmal origin. Clinical neurology and neurosurgery 115:1362-1365

53. Van Calenbergh F, Plets C, Goffin J, Velghe L (1993) Nonaneurysmal subarachnoid hemorrhage: prevalence of perimesencephalic hemorrhage in a consecutive series. Surgical neurology 39:320-323

54. Walcott BP, Stapleton CJ, Koch MJ, Ogilvy CS (2015) Diffuse patterns of nonaneurysmal subarachnoid hemorrhage originating from the Basal cisterns have predictable vasospasm rates similar to aneurysmal subarachnoid hemorrhage. Journal of stroke and cerebrovascular diseases : the official journal of National Stroke Association 24:795-801

55. Whiting J, Reavey-Cantwell J, Velat G, Fautheree G, Firment C, Lewis S, Hoh B (2009) Clinical course of nontraumatic, nonaneurysmal subarachnoid hemorrhage: a single-institution experience. Neurosurgical focus 26:E21

56. Woodfield J, Rane N, Cudlip S, Byrne JV (2014) Value of delayed MRI in angiogram-negative subarachnoid haemorrhage. Clinical radiology 69:350-356

57. Yu D-W, Jung Y-J, Choi B-Y, Chang C-H (2012) Subarachnoid Hemorrhage with Negative Baseline Digital Subtraction Angiography: Is Repeat Digital Subtraction Angiography Necessary? Journal of Cerebrovascular and Endovascular Neurosurgery 14:210-216

58. Zhong W, Zhao P, Wang D, Li G, Sun H, Chen H, Huang S, You C (2014) Different clinical characteristics between perimesencephalic subarachnoid hemorrhage and diffuse subarachnoid hemorrhage with negative initial angiography. Turkish neurosurgery 24:327-332. doi:<https://dx.doi.org/10.5137/1019-5149.JTN.7253-12.1>
